# Genome-Wide Association Study on Grain Length and Grain Width of Rice

**DOI:** 10.3390/biology15010050

**Published:** 2025-12-28

**Authors:** Xing Li, Siyu Wang, Siyuan Ma, Siqi Liu, Wuzhong Yin, Liang Xu, Chiyu Wang, Xiaoqing Yang, Xin Gu, Xunchao Xiang, Yungao Hu

**Affiliations:** 1 Rice Research Institute, Southwest University of Science and Technology, Mianyang 621010, China; weixi305791@163.com (X.L.); xuliang@swust.edu.cn (L.X.);; 2 State Key Laboratory of Crop Gene Resources and Breeding, Beijing 100081, China

**Keywords:** rice, grain shape, GWAS, candidate genes, haplotype

## Abstract

The morphology of rice grains represents one of the most vital agronomic characteristics, significantly impacting both grain productivity and the subsequent milling and nutritional quality of the crop. A comprehensive understanding of the genetic basis and molecular drivers of grain shape is vital for the targeted breeding of high-performance rice lines with consistent yield stability. To pinpoint the genomic regions influencing grain dimensions, we conducted a genome-wide association analysis across a panel of 231 distinct rice accessions, focusing on the discovery of loci associated with length and width. Our analysis revealed four consistent quantitative trait loci (QTLs) distributed across chromosomes 3, 4, and 11. Notably, grain length was associated with qGL3.1, qGL3.2, and qGL11. The first two were co-localized with *GS3* and *SMG3*, respectively, whereas qGL11 likely constitutes a novel locus. One QTL, qGW4, which governs grain width, was found to co-localize with the gene *OsOFP14*. Haplotype analysis further revealed that the characteristic haplotypes of the candidate genes for qGL3.1, qGL3.2, and qGW4 were enriched in eight germplasm accessions (including Newbonnet, Skybonnet, and Lemont), all of which exhibit a slender-grain phenotype. This finding suggests that the specific combination of these characteristic haplotypes is a common genetic signature of slender-grain rice, serving as a potential gene combination for the targeted improvement of rice grain shape. Our results reveal valuable QTLs and candidate genes and highlight specific germplasm resources that can be readily applied in marker-assisted breeding to improve rice grain shape.

## Simple Summary

Rice grain morphology, especially grain shape, is one of the major indicators determinant of yield potential and is controlled by an interconnected gene regulatory network. It also affects rice processing and quality, which are critical for market competitiveness. To achieve higher and more stable rice yields, it is crucial to identify and utilize favorable genetic resources associated with grain shape, and to understand the underlying mechanisms of these genes. Therefore, investigating the genetic regions responsible for rice grain shape is of great importance for both scientific research and rice breeding. In this study, 231 different rice varieties were grown under two different nitrogen conditions, and their grain shapes were measured at maturity. The grain shape data were then correlated with the varieties’ genomic information to identify regions controlling grain shape, along with potential genes and their variants in those regions. Our findings highlight valuable rice accessions and beneficial gene variants enabling breeders to more efficiently generate rice lines exhibiting the desired grain shapes.

## 1. Introduction

Grain shape in rice (*Oryza sativa*) is commonly quantified by grain length, width, and thickness [1]. Variation in these dimensions influences per-plant yield and is also tightly linked to milling performance and the commercial value of rice products [2]. Moreover, grain shape is associated with rice quality: differences in grain size can alter starch accumulation and the distribution of storage proteins, which in turn affects the texture and palatability of cooked rice. Therefore, optimizing grain shape contributes not only to yield improvement but also to quality enhancement and value addition [3]. Genetically, grain shape is a polygenic quantitative trait controlled by multiple loci. To date, numerous grain-shape-related QTLs/genes have been reported [4,5,6], including over 600 QTLs, and nearly 200 loci/genes across all 12 chromosomes have been cloned and functionally validated [7]. Collectively, these advances have deepened our understanding of the genetic regulatory network underlying grain morphology and have provided useful targets and resources for breeding. Continued identification of elite germplasm carrying favorable haplotypes at multiple grain-shape loci, together with mechanistic investigation, should further expand genetic resources for breeding high-yield, high-quality rice.

The genetic control of rice grain reflects the coordination of several signaling modules, among which heterotrimeric G proteins constitute a central node. By alternating between guanosine triphosphate (GTP) bound and GDP bound states through guanosine triphosphatase (GTPase) activity, these proteins transmit signals downstream of membrane-associated pathways and link signaling to metabolic outputs. A well-studied example is *GS3*, which was first mapped by Fan et al. [8]. *GS3* has since become a prominent target for rice breeding. Beyond the role of *GS3*, Sun and colleagues [9] established that the functional divergence among three rice G protein γ subunits—namely *DEP1*, *GGC2 and GS3*—arises from sequence variations in their C-terminal domains. These C-terminal regions of *DEP1* and *GGC2* are crucial for G protein signal transduction, given that their biological activity is contingent upon forming complexes with both the β subunit (RGB1) and the α subunit (RGA1) of the G protein. Although *GS3* does not directly promote grain enlargement, it restricts longitudinal grain growth by outcompeting *DEP1* or *GGC2* for binding to the Gβ subunit, which ultimately limits grain size. Furthermore, the involvement of phytohormones—specifically brassinosteroids (BRs) and cytokinins (CKs)—underscores the critical integration of hormonal pathways in determining grain morphology.

A further noteworthy gene, *SMG3*, was uncovered through the analysis of a cross between the *japonica* accession M494 and the *indica* line Zhong 9B. Research indicates that *SMG3* exerts a negative regulatory effect on the longitudinal and transverse dimensions of rice grains. Subsequent analysis by Li and colleagues [10] further investigated *SMG3* and revealed that the protein it encodes is homologous to the *Arabidopsis* ubiquitin-conjugating enzyme UBC32. This protein interacts with *DGS1* (another grain-size regulatory factor) and facilitates the ubiquitination of BR receptors, consequently modulating the BR signaling cascade, which in turn determines the final dimensions of the grain. In addition to the mechanisms described above, other molecular factors contribute to the development of rice grain shape. Overall, the regulation of rice grain development is a multifaceted process involving various signaling pathways that collectively influence final grain shape.

In study on QTLs related to rice grain morphology, most of the loci are still located in wide chromosome segments, resulting in low resolution. Restricted by technical bottlenecks, previous studies have focused on the analysis of single genes and their regulatory networks, while the exploration of the combined effects of different excellent haplotypes is relatively scarce. In view of this, it is of great theoretical and practical value to explore more QTLs regulating rice grain morphology. In addition, breeders can use the cloned grain morphology regulatory genes to carry out targeted screening of germplasm resources, excavate excellent haplotypes, and further analyze the regulatory effect of major locus pyramiding on grain morphology, so as to provide theoretical support and technical path for molecular improvement of grain morphology-related traits.

To advance these efforts, a particularly valuable yet underexplored approach is to integrate the discovery of novel loci with the systematic evaluation of haplotype combinations—encompassing both established genes and new candidates—within a single, diverse germplasm panel. Such an integrated strategy can directly bridge genetic discovery with breeding application by pinpointing accessions that harbor optimal combinations of alleles. To address this, we implemented a GWAS approach on a panel of 231 rice varieties representing a broad spectrum of global genetic diversity. Moving beyond mere locus identification, we placed special emphasis on haplotype analysis of candidate regions and, crucially, on evaluating the combined effects (pyramiding) of favorable haplotypes. We anticipated that this integrated approach would not only help uncover novel genetic factors but, more importantly, identify specific germplasm accessions carrying elite haplotype combinations for grain shape, thereby providing practical genetic resources for breeding.

## 2. Materials and Methods

### 2.1. Genetic Resources and Experimental Layout

In the present research, we utilized 231 rice germplasm accessions collected worldwide, classified into two subspecies: xian (XI, *indica*) and geng (GJ, *japonica*). These accessions originated from 23 countries and regions, mainly in tropical and subtropical zones (Table S1). The field experiment was conducted in Qinglian, Sichuan Province, China, in May 2024. Two nitrogen (N) treatments were applied: low nitrogen (0 kg ha^−1^, denoted R1) and medium nitrogen (60 kg ha^−1^, denoted R2), using a compound fertilizer from Jinzhengda (15% each of N, P_2_O_5_ and K_2_O). For every accession, a three-row plot was established, with each row consisting of ten individual plants. Phosphorus fertilizer (P_2_O_5_, 60 kg ha^−1^) was applied as a basal dose during soil preparation before transplanting. The planting spacing was 15.5 cm × 20 cm, and all other field management practices followed standard conditions.

### 2.2. Phenotypic Evaluation

After maturation, five representative and independent plants within each accession were randomly selected as biological replicates and harvested individually. Following the approach described by Si et al. [11], the harvested grains were naturally air-dried prior to the evaluation of grain shape-related traits. For each individual plant sample, about 50 plump and uniform grains were randomly selected as a measurement sample. The longitudinal and transverse dimensions, specifically grain length (GL) and width (GW), were quantified for each grain using a Wanshen digital seed imaging system (Model SC-G, Wseen Technology Co., Ltd., Hangzhou, China; http://www.wseen.com/, data retrieved on 4 November 2024) (Figure S1). The final phenotypic value for each accession under a specific nitrogen treatment was calculated as the mean of the trait data obtained the five biological replicates. All operations followed the instrument’s user manual.

### 2.3. Quantitative Assessment of Experimental Data

#### 2.3.1. Phenotypic Evaluation and Data Modeling

The raw grain length and grain width data for all accessions were processed using SPSS (version 22.0). To characterize the distributional features of grain shape traits, we performed descriptive statistical analyses on each trait using the dedicated descriptive statistics function in SPSS, with key metrics including the minimum, maximum, mean, standard error, coefficient of variation, skewness, and kurtosis calculated for each trait. Correlations among rice grain size-related phenotypes were then assessed in RStudio (v2024.04.2+764).

#### 2.3.2. Genetic Structure Analysis

High-density SNP genotypic data for the 231 accessions were extracted from the 3K-RGP (3K Rice Genome Project), specifically utilizing a high-density collection of approximately 4.8 million single-nucleotide polymorphisms (SNPs) [12], a public rice genetic variation database. The final SNP panel was obtained by employing PLINK (version 1.9) [13] to filter the raw data; specifically, we omitted any markers with a missing rate > 10% or those failing to meet the frequency requirements (MAF > 5% and major allele frequency < 95%). Population structure was inferred via ADMIXTURE (1.3.0) [14], and the derived ancestry component matrix (q) was retained for subsequent downstream analyses. Kinship (K) among the 231 accessions was estimated using TASSEL (version 5.0) [15], with all high-quality SNPs integrated to construct the pairwise relationship matrix. Furthermore, principal component analysis (PCA) was conducted to supplement the assessment of population genetic stratification, and the obtained PCA scores, combined with the kinship matrix (K), were incorporated as covariates in the subsequent analysis.

#### 2.3.3. Identification of Grain-Shape Loci via GWAS

Using the filtered high-quality SNP dataset, we performed a genome-wide association analysis by implementing a mixed linear model (MLM) in the TASSEL software (5.0) platform. We set a significance threshold of *p* < 1 × 10^−4^. If two or more significant SNPs were found within 200 kb of each other, they were considered to represent a single QTL region. For each identified QTL region, we compared the R^2^ values of all significant SNPs and used the highest R^2^ as the explained variance (contribution rate) for that QTL. Manhattan and Q–Q plots were produced in R with the qqman package (4.4.1), while linkage disequilibrium (LD) patterns were visualized using LDBlockShow (3.1.1).

#### 2.3.4. Identification of Candidate Genes and Haplotype Characterization

Based on gene annotations from the Rice Annotation Project Database (RAP-DB), candidate regions for each QTL were delineated as a 100 kb window flanking the peak SNP. Hypothetical proteins, transposable elements, and pseudogenes were excluded from consideration [16]. Within these intervals, any genes homologous or functionally similar to known grain-shape genes were initially flagged as candidate genes (using the Rice Expression Database for guidance). Subsequently, we retrieved SNP data from the Rice Genomic Variation and Functional Annotation Database (IC4R Varmap) and PLINK to extract all non-synonymous SNPs within each candidate gene, including variants in the 2 kb upstream promoter region, coding exons, introns, and the 1 kb downstream region. To ensure sufficient polymorphism, SNP loci with a major allele frequency >80% or a missing data rate >5% were discarded. We then conducted a haplotype analysis of the remaining variant sites using Haploview 4.2 [17] defining haplotypes according to LD patterns. After performing multiple comparisons of trait values among the different haplotypes, genes exhibiting significant phenotypic variation among haplotypes were retained as the final set of candidate genes [18].

#### 2.3.5. Pyramiding Analysis of Excellent Haplotypes of Grain Shape Genes in Germplasms

Initially, IBM SPSS Statistics 22 was used to compute the mean and standard deviation of grain length and grain width across the germplasm panel. Subsequently, multiple comparison analyses were conducted to evaluate differences in these traits among distinct haplotype combinations of grain-shape genes, with the aim of identifying accessions exhibiting favorable grain morphology.

## 3. Results

### 3.1. Phenotypic Characterization

**Figure 1:**
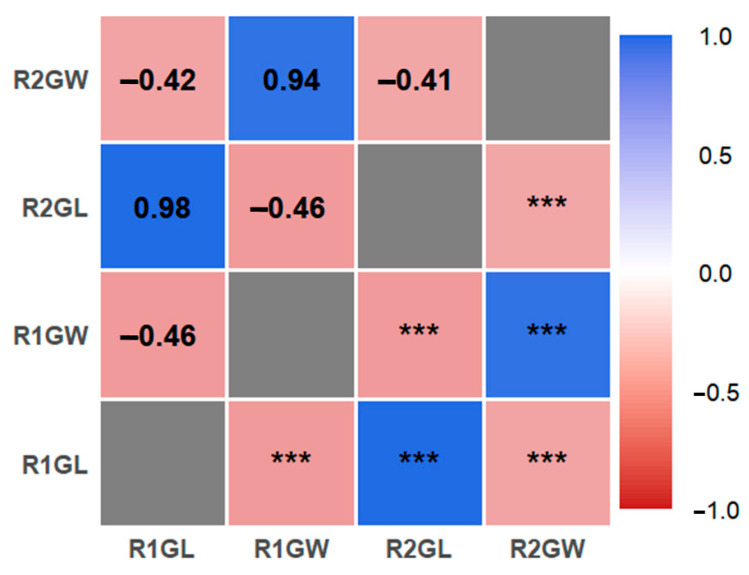
Correlation analysis of 231 rice grain size traits. The color gradient corresponds to the strength and direction of Pearson correlation coefficients (ranging from −1.0 to 1.0; blue indicates positive correlation, red indicates negative correlation), and “***” denotes a statistically significant correlation at the *p* < 0.001 level. R1 and R2 represent two independent experimental environments; GL represents grain length, and GW represents grain width.

The 231 rice accessions used in this study were collected from 23 regions (Table 1). Evaluation of grain length and width across the germplasm population indicated that under the R1 environment, the ranges were 6.30–11.81 mm for length and 2.15–3.76 mm for width, and under the R2 environment the ranges were 6.11–11.40 mm and 2.11–3.71 mm, respectively (Table 2). The coefficients of variation for both traits were similar in the two environments, indicating comparable population distributions and no significant overall difference due to the nitrogen treatment. These results suggest that grain shape in this germplasm population is genetically stable and only slightly influenced by the different nitrogen levels. Under the R1 condition, the grain length had kurtosis and skewness values of 0.05 and 0.21, respectively (mean length 8.47 mm), and grain width had kurtosis and skewness of 0.68 and 0.12 (mean width 2.90 mm). Under R2, the grain length kurtosis and skewness were 0.12 and 0.21 (mean length 8.44 mm), and for grain width they were 0.55 and 0.20 (mean width 2.89 mm). In both environments, both grain length and grain width exhibited absolute skewness and kurtosis values below 1, indicating that these grain shape traits follow an approximately normal distribution (consistent with their quantitative, polygenic control). The genetic characteristics of this rice germplasm population therefore meet the statistical assumptions for performing a GWAS. Correlation analysis revealed the relationships among grain size-related traits of the 231–rice natural population are shown in Figure 1. Grain length and grain width were strongly and negatively correlated in both the R1 and R2 environments. Accordingly, genome-wide association analysis of grain-shape traits is justified.

### 3.2. Population Structure Analysis

**Figure 2:**
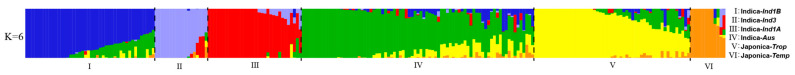
Admixture analyses of the population structure of 231 rice germplasms. Cluster analysis results of population genotypes, in which each color represents a group.

Population structure analysis revealed the genetic stratification characteristics of the 231 rice germplasms. Cross-validation error assessment showed that the lowest error value was obtained when the number of ancestral groups (K) was set to 6 (Figure 2), indicating that these rice germplasms can be optimally divided into six distinct ancestral groups. This stratification result is visually presented in the ADMIXTURE cluster plot (Figure 2), where each color represents one ancestral group, and the color proportion in each individual germplasm reflects the genetic component contribution of the corresponding ancestral group.

### 3.3. Genome-Wide Association Study on Rice Grain Shape

**Figure 3:**
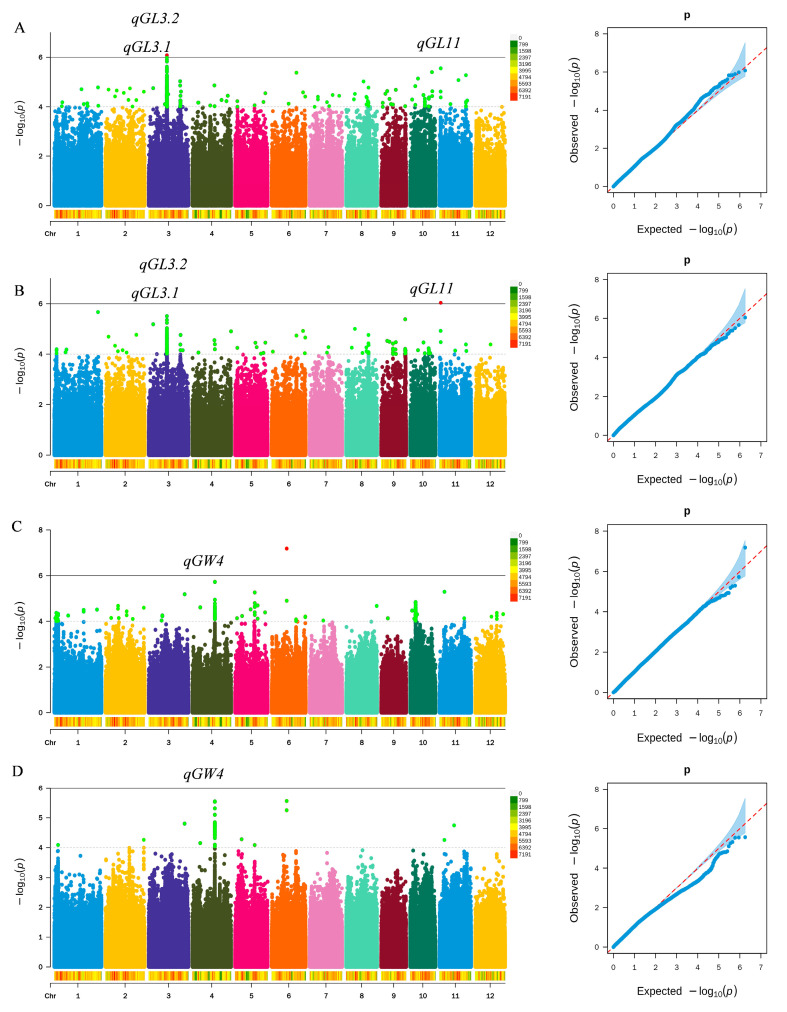
Genome-wide association results for rice grain length and grain width. (**A**) Manhattan and Q–Q plots for grain length under the R1 environment. (**B**) Manhattan and Q–Q plots for grain length under the R2 environment. (**C**) Manhattan and Q–Q plots for grain width under the R1 environment. (**D**) Manhattan and Q–Q plots for grain width under the R2 environment.

Genome-wide association studies targeting grain length and grain width were performed using the rice germplasm panel, with the corresponding results shown in Figure 3. In total, four QTLs associated with grain shape were detected on chromosomes 3, 4, and 11 (Table 3). Consecutive significant SNPs (two or more within ≤200 kb) were considered a single QTL locus. Based on this criterion, four QTLs were detected, including three loci influencing grain length and a single locus affecting grain width. Among the identified loci, qGL3.1, qGL3.2, and qGL11 were consistently detected for grain length across both R1 and R2 environments. Notably, qGL11 is a novel grain length QTL on chromosome 11, accounting for 15.07% of phenotypic variation, with a physical interval of 1,113,362–1,113,774 bp. qGL3.1 explains 14.19% of variation and lies at 16,729,325–16,832,795 bp; it co-localizes with the cloned grain-length gene *GS3*, suggesting that *GS3* is the gene underlying qGL3.1 [8]. Likewise, qGL3.2 explains 11.32% of the variation and is located at 16,878,251–16,893,486 bp; it co-localizes with the cloned gene *SMG3*, indicating that *SMG3* is the likely candidate gene for qGL3.2 [6]. Regarding grain width, the QTL qGW4 was detected under both R1 and R2, explaining 13.03% of the variation and located at 20,599,993–20,620,638 bp on chromosome 4. This region co-localizes with *OsOFP14*, suggesting that *OsOFP14* is the candidate gene underlying qGW4 [19].

### 3.4. Prediction of Candidate Genes for Grain Length QTL and Their Haplotype Analysis

**Figure 4:**
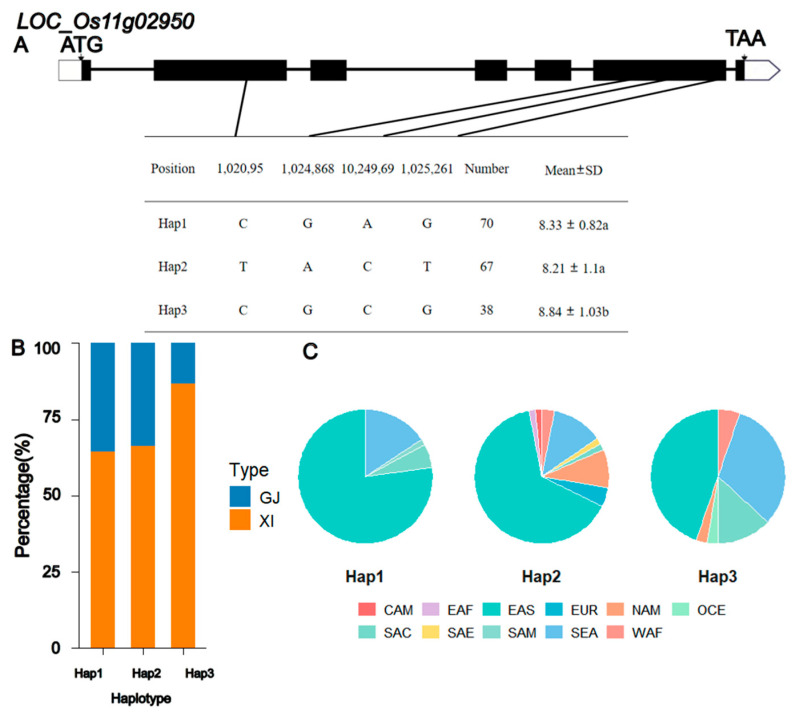
Analysis of the *LOC_Os11g02950* haplotype. (**A**) Schematic representation of structure and haplotypes. Data are expressed as mean ± standard deviation. Values followed by different lowercase letters (a, b) in the figure indicate significant differences at *P* <0.05 (tested by Duncan’s multiple range test) (**B**) The subpopulation composition of *LOC_Os11g02950*. (**C**) Geographical distribution of different haplotypes of *LOC_Os11g02950*.

Analysis of the qGL11 interval (which contains 34 annotated genes) was conducted after excluding hypothetical proteins, transposons, and pseudogenes. By screening for genes potentially involved in grain shape pathways, we identified two candidate genes associated with grain length in this interval (see Supplementary Table S2). Their annotations are: a putative glycosyltransferase family 8 member (*LOC_Os11g03160*) and a putative auxin efflux carrier protein *(LOC_Os11g02950*). Further significance analysis of grain length among the haplotypes of these two genes revealed that *LOC_Os11g03160* had two major haplotypes, with no significant difference in grain length between them (Figure S2). In contrast, *LOC_Os11g02950* had three major haplotypes, and the grain length of Hap3 was significantly higher than that of the other two haplotypes (Figure 4). Compared with *LOC_Os11g03160*, the differences in grain length among the haplotypes of *LOC_Os11g02950* were more pronounced, suggesting that *LOC_Os11g02950* might be a candidate gene for qGL11. However, further validation is required to confirm this conclusion.

For qGL3.1, the analysis confirmed that *GS3* (*Os03g0407400*) is the candidate gene, as this locus lies in a strong LD block containing *GS3* (Figure S3A). We identified two major haplotypes of *GS3* based on the known functional SNP (a C→A mutation causing a 178–amino acid truncation in the *GS3* protein [8]; Figure S3B). Grain length differed significantly between the haplotypes: *GS3*^Hap1^ had a greater mean length (8.62 mm) than *GS3*^Hap2^ (8.33 mm; *p* < 0.05). *GS3*^Hap1^ was carried by 132 accessions (mostly *indica*, primarily from East Asia), whereas *GS3*^Hap2^ was found in 99 accessions (mainly *japonica*, with a high proportion from East and Southeast Asia) (Figure S3B–D).

**Figure 5:**
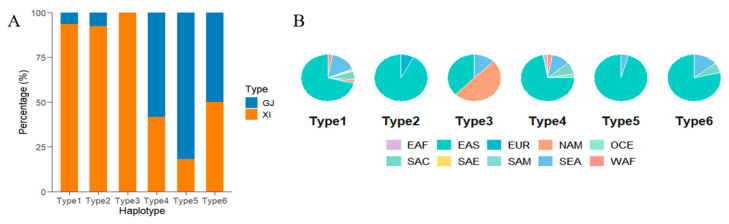
Germplasm analysis of 6 aggregation types of grain length and grain width gene haplotypes. (**A**) Composition of subgroups for each aggregation type. (**B**) Geographical distribution of germplasm in various aggregation types.

For the qGL3.2 locus, a 100 kb flanking region around the significant SNP (Chr3: 16,888,255 bp) was defined as the candidate interval. Linkage Disequilibrium analysis showed that the candidate gene *SMG3* (*Os03g0410000*) lies within a region of high SNP linkage (Figure S4A). By analyzing a combination of three variants within the 5′ untranslated region (UTR) and three within the coding sequence of *Os03g0410000*, we successfully distinguished two primary haplotypes, as illustrated in Figure 5B. Grain length also differed significantly between *SMG3*^Hap1^ and *SMG3*^Hap2^ (*p* < 0.05), with *SMG3*^Hap1^ showing a higher mean grain length (8.47 mm) than *SMG3*^Hap2^ (*p*< 0.05). Considering the haplotype associated with longer grain as favorable, *SMG3*^Hap1^ was designated as the advantageous haplotype for *SMG3*. *SMG3*^Hap1^ was predominantly represented by *indica* varieties (Figure S4C), and accessions carrying this haplotype were mainly distributed across Asian countries (Figure S4D).

### 3.5. Prediction of Candidate Genes for Grain Width QTL and Their Haplotype Analysis

For qGW4, the candidate interval (Chr4: 20,620,638 bp ± 100 kb) contained *OsOFP14* (*Os04g0415100*) in a high-LD segment (Figure S5A). Based on a trio of single-nucleotide polymorphisms located within the *OsOFP14* coding sequence, the population was classified into three predominant haplotypes (illustrated in Figure S5B). One haplotype, *OsOFP14*^Hap3^ (defined by the TAA allele combination), was associated with a mean grain width of ~2.70 mm (Figure S5B). This haplotype occurred predominantly in indica accessions (Figure S5C), and the accessions carrying *OsOFP14*^Hap3^ were mainly from North America and East Asia (Figure S5D).

### 3.6. Aggregation Analysis of Different Haplotypes of Grain Shape Genes in Germplasm Resources

Further analysis of genotype–phenotype patterns indicated an epistatic interaction between *GS3* and *OsOFP14*: *OsOFP14* influenced grain width only when *GS3* was nonfunctional. This observation is consistent with the genetic pattern in which a major-effect gene masks the effect of a minor gene in a multi-locus context. In contrast, *SMG3* did not have a significant effect on grain shape in our study. Li et al. [10] have noted that the protein product of *SMG3* is an E2 ubiquitin-conjugating enzyme associated with the ERAD pathway; it necessitates a physical association with *DGS1* to modulate BR signaling and dictate the longitudinal growth of grains. The lack of a detectable *SMG3* effect here may be due to a uniformly functional DGS1 in our population, whereas *SMG3*’s influence was apparent in other genetic backgrounds, where DGS1 allowed its regulatory potential to be expressed. Among the six major haplotype combinations of the three genes, Types 1, 2, and 3 had significantly greater grain length than Types 4, 5, and 6 (*p* < 0.05). Among the categorized groups, Type 3 accessions were characterized by the most substantial longitudinal growth, averaging 8.98 mm. Conversely, Type 5 exhibited the minimum average grain length at 7.19 mm, though it was distinguished by the highest mean grain width (3.27 mm), as detailed in Table 4. This pyramiding analysis thus identified a “slender-grain” haplotype combination (Type 3)—exemplified by accessions like Newbonnet, Skybonnet, and Lemont—which are predominantly indica varieties from North America. Conversely, the “short-round” haplotype combination (Type 5), e.g., Kosh, Kongyu 131, Jia 33, was mainly found in japonica varieties from Asia (Figure 5A,B).

## 4. Discussion

As a primary determinant of harvestable biomass and commercial suitability, grain shape in rice serves as an essential parameter for optimizing yield and industrial processing efficiency [20]. Specifically, grain width is positively correlated with average chalkiness, whereas the length–width ratio presents a negative correlation with chalkiness degree and slender rice grains, featured by transparency and lack of chalky texture, thus possess substantial commercial value in the global marketplace [21,22]. Elucidating the molecular underpinnings that govern grain architecture is indispensable for developing targeted selection programs intended to optimize rice grain dimensions. Our grasp of the genetic factors influencing rice grain traits has been substantially enhanced by the results of recent high-throughput association studies. Chen et al. [23] performed GWAS on 280 japonica rice accessions and identified 15 grain shape-related QTLs. Wang et al. [24] identified 61 QTLs using 265 natural populations, and their discovery of monosaccharide transporter gene *LOC_Os03g39710* as a grain length regulator lends further support to the possibility that chromosome 3 may harbor key loci for grain shape control. By conducting a genome-wide association analysis on a diverse panel of 231 rice lines, we successfully mapped several major-effect loci governing grain architecture, specifically qGL3.1, qGL3.2, qGL11, and qGW4. These results expand the existing framework of rice grain morphogenesis and offer practical genomic targets for the precision breeding of varieties with optimized physical dimensions.

Identification of the qGL11, a novel grain-length QTL, opens up new possibilities for improving grain size in rice. In contrast, qGL3.1 and qGL3.2 co-localize with the known genes *GS3* and *SMG3*, respectively, and our findings validate their roles in a new genetic background, providing additional evidence of their importance in shaping rice grain morphology. Notably, we also detected qGW4, which co-localizes with *OsOFP14*, a gene known to regulate grain width. These observations imply a degree of mechanistic crosstalk between the genetic networks governing the longitudinal and transverse dimensions of the rice grain.

Despite these advances, several questions remain. For instance, the qGL11 region may harbor additional gene(s) with major effects on grain length, warranting further fine mapping and functional validation. Moreover, interactions between these QTLs and environmental factors (as highlighted by comparisons with other studies) emphasize the complexity of grain shape regulation. This complexity is further reflected in the differential effects of grain shape QTLs across genetic backgrounds, as demonstrated by our Type 3 vs. Type 5 haplotype comparisons (Figure 5).

Although the targeted mutagenesis of pivotal grain morphology determinants, such as *GS3* and *SMG3,* has been effectively achieved through CRISPR/Cas9 technology [25,26], our study highlights the complementary potential of pyramiding multiple QTLs to achieve desirable grain shapes. The identification of accessions with extremely slender grains (Type 3 combination) versus very short, round grains (Type 5 combination) underscores the possibility of marker-assisted selection for specific grain morphologies, an approach that could accelerate breeding programs. However, the effectiveness of this pyramiding strategy will depend on fine-tuning haplotype combinations—notably, some theoretically possible haplotype combinations were not observed in our population, suggesting limitations in available diversity.

In a large-scale association mapping effort, six quantitative trait loci (QTLs) (e.g., qTGW3.1, qTGW9, qGL4/*qRLW4*) governing grain morphology were localized by Niu and colleagues [27], who leveraged a massive panel of 2453 lines from the 3K-RGP. A comparison between their results and ours revealed no overlapping loci. This suggests that the detection of grain shape QTLs is strongly influenced by environmental conditions and the genetic composition of the population. In other words, the specific plant materials used in a GWAS can directly affect which QTLs are found, and grain shape QTLs may have effects that are expressed in some genetic backgrounds but not in others. Indeed, the choice of plant materials can strongly influence GWAS results, as different genetic backgrounds may allow different QTLs to be detected. For example, through the use of an M494 × Zhong 9B cross mapping population, Li and colleagues [6] localized *SMG3* as a multifaceted QTL that influences grain dimensions, weight, and panicle density. Functionally, *SMG3* acts by stimulating the growth and division of glume cells, which imposes a negative constraint on grain size while simultaneously enhancing the total number of grains produced. Notably, *SMG3* maps to the same interval as qGL3.2 in our study, indicating that *SMG3* is likely the causal gene for qGL3.2 (as further supported by our haplotype analysis). According to Zhao et al. [19], the protein *OsOFP14* functions as an antagonist to *GS9.* It integrates into a larger molecular assembly alongside *OsOFP8* and *OsGSK2,* thereby modulating the orientation or frequency of glume cell division to dictate final grain morphology. Together with our evidence (from haplotype analysis) suggesting *OsOFP14* is the gene underlying qGW4, this finding reinforces the credibility of the QTLs we identified. Additionally, our identification of two candidate genes related to seed morphology near the significant Chr11 SNP (position ~1.113 Mb) suggests the presence of a high-priority candidate gene for longitudinal grain development that demands additional empirical scrutiny. This finding reaffirms the conclusion reported by Wang et al. [28], namely that synthesizing data from haplotype block frameworks and functional gene characterizations can effectively streamline the identification of candidate genes, thereby providing a more targeted approach for elucidating the molecular architecture that underlies multifaceted grain characteristics.

Varietal improvement is a key means of incorporating beneficial germplasm traits into elite cultivars. However, conventional breeding is time-consuming, labor-intensive, and not always effective when it comes to achieving specific trait outcomes. Yang et al. [29] found that pyramiding known grain shape genes can enable precise improvement of grain characteristics. Similarly, Xia et al. [30] reported an epistatic interaction between *GL3.3* and *GS3*–pyramiding these two genes in one genetic background produced a significant increase in milled grain morphology.

In the present research, the combinatorial effects of different haplotypes at qGL3.1 (*GS3*), qGL3.2 (*SMG3*), and qGW4 (*OsOFP14*) were examined. The Type 3 haplotype combination (*GS3*^Hap1^ + *SMG3*^Hap1^ + *OsOFP14*^Hap3^) resulted in reduced grain width but increased grain length–producing a slender grain phenotype. In contrast, the Type 5 combination (*GS3*^Hap2^ + *SMG3*^Hap1^ + *OsOFP14*^Hap2^) led to shorter grain length and greater grain width, yielding a short, round grain. From these groups, we identified eight accessions with slender grains and 22 accessions with short-round grains. These genetic resources are valuable for grain shape improvement and could be directly utilized to shorten the breeding cycle via marker-assisted breeding.

However, the effectiveness of pyramiding these QTLs for grain shape improvement depends on fine-tuning haplotype combinations across different genetic backgrounds [31]. The differential effects of these QTLs observed among rice accessions suggest that genetic background plays a significant role in breeding outcomes. These findings underscore the imperative to investigate how genomic context modulates grain morphology, especially within the framework of large-scale marker-based selection or precision genome engineering Via CRISPR.

In addition to the limitation of genetic background, the breeding improvement of rice grain shape traits is further restricted by its internal development and physiological characteristics. The lemma and palea of rice play a role as a sink structure, which determines the maximum spatial volume of caryopsis development, and the formation of endosperm is not only limited by the size of this sink, but is also closely related to the filling process such as photosynthetic product transport and starch accumulation efficiency [32]. This double limitation means that in breeding programs, the aggregation of favorable endosperm QTLs does not guarantee the expected yield and quality gains, because under specific cultivation conditions, the glume shape may not be fully matched with endosperm filling. Therefore, future breeding strategies should take this limitation into account and combine sink-related traits (glume size) with source-related traits (grain filling capacity).

Additionally, although our study identified key haplotypes associated with grain shape, the exact molecular mechanisms through which these haplotypes influence grain development remain unclear. Providing definitive proof of these candidate genes’ influence on grain architecture will require targeted empirical strategies, such as loss-of-function mutagenesis and gain-of-function assays. Moreover, investigating how these genes interact with environmental factors (e.g., nutrient availability, temperature, water stress) will provide deeper insight into the stability of grain shape traits under field conditions.

Our findings have significant implications for future rice breeding programs. Identifying specific grain shape haplotypes enables more precise selection of parent lines, thereby accelerating the genetic enhancement of rice lines possessing superior grain attributes. Most existing studies focus on the relationship between single genes and traits; however, as a multifaceted quantitative characteristic, grain shape is dictated by the cumulative activity of various genes distributed across the rice genome. Therefore, conducting multi-gene combinatorial regulation of traits based on GWAS results is more likely to provide effective combination patterns for marker-assisted breeding [33]. Compared with single-marker analysis, haplotype analysis integrates multiple linked genetic variations, which can more accurately reflect the functional differences of genes and improve the reliability of identifying favorable genetic loci related to grain shape. Furthermore, combining these favorable haplotypes with other agronomically important traits (such as disease resistance or drought tolerance) offers opportunities for developing climate-resilient rice varieties. Ultimately, integrating these genetic improvements into breeding pipelines will require a multidisciplinary approach—incorporating genomic selection methods and advanced phenotyping technologies—to ensure that desired traits are effectively realized in new cultivars.

The identification of these grain shape QTLs and their associated haplotypes marks an important step towards improving rice morphology. Beyond elucidating the genomic architecture of grain morphology, this investigation furnishes a practical roadmap for the rapid development of specialized rice cultivars by identifying optimal allelic configurations. Further research into the molecular mechanisms of these QTLs, as well as their interactions with environmental factors, will pave the way for more efficient and sustainable rice breeding strategies.

## 5. Conclusions

In conclusion, a GWAS on grain length and width was conducted in 231 diverse rice accessions, and identified four grain shape QTLs distributed on chromosomes 3, 4, and 11. Notably, qGL11 is a novel grain-length locus with no previously known gene, while qGL3.1, qGL3.2, and qGW4 co-localize with previously characterized genes (*GS3*, *SMG3*, and *OsOFP14,* respectively). Through haplotype combination analysis of both established genetic regulators of grain morphology and new candidate genes, we identified eight accessions with slender grains and 22 accessions with short, round grains. These specific accessions constitute a vital germplasm pool for elite rice cultivation, paving the way for the targeted enhancement of grain architecture in subsequent breeding cycles.

## Figures and Tables

**Table 1:** Distribution of rice germplasm accessions used in this study.

Nation	Number	Nation	Number	Nation	Number
America	7	India	6	Philippines	27
Australia	1	Indonesia	1	Republic of Korea	1
Bangladesh	1	Italy	2	Senegal	1
Brazil	1	Japan	4	Spain	1
China	160	Madagascar	1	Sri Lanka	1
Colombia	1	Malaysia	2	Thailand	3
Cote d’Ivoire	3	Nepal	1	Vietnam	2
Cuba	1	Pakistan	3		

**Table 2:** Statistical Characterization of Rice Grain Shape Traits.

Traits	Repeat	Mean ± SD/mm	CV%	Kurtosis	Skewness
Grain length	R1	8.47 ± 1.04	12.28%	0.21	−0.05
	R2	8.44 ± 1.03	12.20%	0.21	−0.12
Grain width	R1	2.9 ± 0.34	11.72%	0.12	−0.68
	R2	2.89 ± 0.34	11.76%	0.2	−0.55

Note: Mean ± SD represents the mean value ± standard deviation (unit: mm); CV% denotes the coefficient of variation. Independent samples *t*-test was performed to compare the differences in grain shape traits between two biological repeats (R1 and R2). The results showed no significant differences between R1 and R2 for both grain length and grain width (*p* > 0.05), confirming the reliability and reproducibility of the phenotypic measurement.

**Table 3:** Significant SNPs identified by genome-wide association analysis for rice grain length and grain width.

QTL	SNP	Chr.	SNP Position	*p* Value	R^2^
qGL3.1	3-16729325	3	16,729,325	1.42 × 10^−5^	13.43%
	3-16730184	3	16,730,184	1.99 × 10^−5^	12.26%
	3-16731043	3	16,731,043	4.69 × 10^−5^	12.56%
	3-16731141	3	16,731,141	5.23 × 10^−5^	11.96%
	3-16736632	3	16,736,632	1.54 × 10^−5^	13.37%
	3-16732377	3	16,732,377	6.85 × 10^−6^	14.19%
	3-16732795	3	16,732,795	1.51 × 10^−5^	12.36%
qGL3.2	3-16878251	3	16,878,251	9.95 × 10^−5^	10.55%
	3-16884927	3	16,884,927	7.74 × 10^−5^	10.93%
	3-16888255	3	16,888,255	8.89 × 10^−5^	11.32%
	3-16893486	3	16,893,486	9.99 × 10^−5^	10.62%
qGL11	11-1113362	11	1,113,362	2.79 × 10^−6^	15.07%
	11-1113774	11	1,113,774	4.15 × 10^−5^	11.23%
qGW4	4-20599993	4	20,599,993	3.90 × 10^−4^	8.67%
	4-20608230	4	20,608,230	2.60 × 10^−4^	9.04%
	4-20620638	4	20,620,638	1.16 × 10^−5^	13.03%
	4-20620895	4	20,620,895	2.22 × 10^−5^	12.12%
	4-20620990	4	20,620,990	9.58 × 10^−4^	7.77%

Note: QTL represents Quantitative Trait Locus; SNP denotes Single Nucleotide Polymorphism; Chr stands for Chromosome; *p* Value indicates the significance level; R^2^ represents the phenotypic variance explained (%).

**Table 4:** Evaluation of Haplotype Combinations and Their Impact on Grain Dimensions Under R1 Conditions.

Combination	*GS3*	*SMG3*	*OsOFP14*	Number	Grain Length/mm	Grain Width/mm
Type1	Hap1	Hap1	Hap1	107	8.96 ± 0.84 a	2.78 ± 0.27 a
Type2	Hap1	Hap2	Hap2	13	8.8 ± 0.48 a	2.79 ± 0.21 a
Type3	Hap1	Hap1	Hap3	8	8.98 ± 0.63 a	2.62 ± 0.2 a
Type4	Hap2	Hap1	Hap1	36	8 ± 0.82 b	3.02 ± 0.25 b
Type5	Hap2	Hap1	Hap2	22	7.19 ± 0.76 c	3.27 ± 0.27 c
Type6	Hap2	Hap1	Hap3	14	7.9 ± 0.85 b	2.98 ± 0.28 d

Note: Values are presented as mean ± standard deviation. Different lowercase letters (a–d) in the same column indicate significant differences among combinations at *p* < 0.05 (determined by Duncan’s multiple range test).

## Data Availability

The datasets supporting the conclusions of this article are included within the article and Supplementary Materials.

## Supplementary Materials

The following supporting information can be downloaded at https://www.mdpi.com/article/10.3390/biology15010050/s1, Table S1: The names and geographical origins of 231 rice accessions; Table S2: Candidate genes located within the significant region on chromosome 11. Figure S1. Morphological phenotypic appearance of GL and GW in different plant materials. Figure S2. Analysis of the *LOC_Os11g03160* haplotype. Figure S3. Analysis of the *GS3* haplotype. Figure S4. Analysis of the *SMG3* haplotype. Figure S5. Analysis of the *OsOFP14* haplotype.

## Abbreviations

The following abbreviations are used in this manuscript:

GWAS	Genome-Wide Association Study
QTL	Quantitative Trait Locus
MLM	Mixed Linear Model
SNP	Single Nucleotide Polymorphism
