# Supplementary material for: Genome-Wide Association Study on Grain Length and Grain Width of Rice
Source: Biology (Basel). 2025 Dec 28;15(1):50. doi: 10.3390/biology15010050 (PMC12785118; doi:10.3390/biology15010050)
Supplement: Supplementary file 1 [file biology-15-00050-s001.zip › biology-4038675-supplementary.pdf]

Supplementary material

Table S1. The names and geographical origins of 231 rice accessions.

Number	variety	Source	Number	variety	Source	Number	variety	Source
1	zaodaxian	China	78	IRBB21	Philippines	155	TEBONNET	America
2	heizaogu	China	79	J4155	China	156	THAIPERLA	Thailand
3	2428	China	80	JASMINE85	Thailand	157	TN1	Philippines
4	xikehui2928	China	81	Jaya	India	158	Taihui808	China
5	xikehui7215	China	82	Jia33	China	159	Taiyin1hao	China
6	803B	China	83	Jianghui151	China	160	Taizhongzailai1	China
7	9311	China	84	Jiangxisimiao	China	161	Tetep	Vietnam
8	AUS449	Australia	85	Jiefangxian	China	162	Tumba	Indonesia
9	B1B	China	86	Jin23B	China	163	Tuojiangnuo5hao	China
10	B3B	China	87	Jingxiang1A	China	164	UPRH33	Philippines
11	BG367-4	China	88	K17B	China	165	V20B	China
12	BG94-1	Sri Lanka	89	KALIBORO600	Bangladesh	166	WA_BANG	Republic of Korea
13	Balila	Italy	90	KASALATH	India	167	WSSM	China
14	Basmati1	Pakistan	91	KY131	China	168	Wuyugeng20hao	China
15	Basmati370	Pakistan	92	Katy	America	169	Xiangaizao8hao	China
16	CDR22	China	93	Kosh	Japan	170	Xiangdao	China
17	CHENGHUI425	China	94	L301B	China	171	Xianggu	China
18	CICA8	Colombia	95	LALSAR	Nepal	172	Xiangwanxian5hao	China
19	Changxianggu	China	96	LATSIBOZAKA112-1	Madagascar	173	Xianhui207	China
20	Chenghui149	China	97	LJ	China	174	Xingshi	China
21	Chenghui177	China	98	Leah	America	175	Y3551	China
22	Chenghui448	China	99	Lehui188	China	176	Y58S	China
23	Chuanxiang29b	China	100	Liangeng10hao	China	177	Yahui2115	China
24	D11	India	101	Lijiangxintuanheigu	China	178	Yanhui559	China
25	D297B	China	102	Luhui602	China	179	Yixiang1b	China
26	D62B	China	103	Lunhui422	China	180	YouIb	China
27	DINORADO	Philippines	104	MIANHUI2040	China	181	YuMiDao	China
28	Daohuaxiang2hao	China	105	MIAOZHAN	China	182	Yueguang	Japan
29	Dshanb	China	106	MOROFIN	Cote d'Ivoire	183	Yuehesimiao	China
30	Duoxi1hao	China	107	Malaihong	Malaysia	184	Yuzhenxiang	China
31	Dxiang1b	China	108	Mamagu	China	185	Zhefu802	China

32	Erjiuqing	China	109	Meiguodao	America	186	Zhenhui084	China
33	Fei11	China	110	Mian5	China	187	Zhenshan97b	China
34	Fuhui838	China	111	Mianhui2009	China	188	Zhenzhuai11	China
35	Fuhui9303	China	112	Mianhui523	China	189	Zhong9b	China
36	G46B	China	113	Mianhui528	China	190	aizizhan	China
37	GRITNA	Italy	114	Mianhui725	China	191	changmi011	China
38	GU154	Cuba	115	Minghui63	China	192	chenghui727	China
39	GUI630	China	116	Minkezhan	China	193	dianrui409	China
40	Gu223	China	117	Miyang23	China	194	diantun502	China
41	Guichao13	China	118	Miyang46	China	195	dongtingwanxian	China
42	Guichao2hao	China	119	Miyang70	China	196	erjiunan1hao	China
43	Guiluai8hao	China	120	Miyang84	China	197	gaoliangdao	China
44	Gumei2	China	121	N22	India	198	gui99	China
45	H52	China	122	NERICA-L-27	Cote d'Ivoire	199	heigu	China
46	HIRAKAWA_OKUTE	Japan	123	NamRoo	Philippines	200	heinuo	China
47	Haobayong1	China	124	Nangeng11	China	201	hexi41	China
48	HongmeizaoB	China	125	Nangeng44	China	202	huke3hao	China
49	HuangJinQing	China	126	Nanhui511	China	203	jiegunuo	China
50	Huazhan	China	127	Nanjing11	China	204	meigui1hao	China
51	Huhui17	China	128	Nanjing16	China	205	mian2A	China
52	Huhui602	China	129	Newbonnet	America	206	minghui69	China
53	IAC47	Brazil	130	Ninggeng1hao	China	207	shengtai1hao	China
54	II32B	China	131	PADI_ADONG_DUMARAT	Malaysia	208	shuhui162	China
55	IR1544-181-1-1	China	132	PAGAIYAHAN	China	209	shuhui527	China
56	IR2071-77-9-3-5	Philippines	133	PULUTAN	Philippines	210	shuhui881	China
57	IR2153-26-3-5-6	Philippines	134	Peiai64	China	211	suyunuo	China
58	IR24	Philippines	135	Qimiaoxiang	China	212	wanhui88	China
59	IR25588-7-3-1	Philippines	136	Qingke	China	213	yihui1313	China
60	IR25924-51-2-3	Philippines	137	Qingxiaojinzao	China	214	yihui1577	China
61	IR25926-14-3-2	Philippines	138	Qishanzhan	China	215	yihui3003	China
62	IR26	Philippines	139	R130	China	216	zhaiyeqing8hao	China
63	IR28	Philippines	140	R162	China	217	zhenlong13	China
64	IR29	Philippines	141	R325	China	218	zhonghua11	China
65	IR30	Philippines	142	R8006	China	219	zhongxiang1hao	China
66	IR42	Philippines	143	Ribenqing	Japan	220	zimi	China
67	IR44	Philippines	144	SAL_BUI_BAO	Vietnam	221	5228	China
68	IR50	Philippines	145	SANA	India	222	5252	China
69	IR56	Philippines	146	SENIA	Spain	223	CG14	Senegal

70	IR58	Philippines	147	SKYBONNET	America	224	CN1	China
71	IR64	Philippines	148	SOLAY_GHAT	Pakistan	225	COX21	China
72	IR661	Philippines	149	SPR7284-57-5	Thailand	226	DG	China
73	IR665	Philippines	150	Shuanggui1hao	China	227	FS32	China
74	IR77298-14-1-2-10	Philippines	151	Shuhui498	China	228	G8	China
75	IR8	Philippines	152	Shuhui548	China	229	Lemont	America
76	IRAT144	Philippines	153	Shuiyuan317	China	230	R28	China
77	IRAT36	Cote d'Ivoire	154	T21	India	231	TM	China

Table S2. Candidate genes located within the significant region on chromosome 11.

MSU_Locus	Gene name or annotation
LOC_Os11g02950	**auxin efflux carrier component, putative, expressed**
LOC_Os11g02964	FAR1 family protein, expressed
LOC_Os11g03050	cytidylyltransferase domain containing protein, expressed
LOC_Os11g03060	ESCRT-III components
LOC_Os11g03070	ATCHX, putative, expressed
LOC_Os11g03110	GRAS transcription factor
LOC_Os11g03130	E2F-related protein, putative, expressed
LOC_Os11g03160	**glycosyl transferase, family 8, putative, expressed**
LOC_Os11g03200	EMB2748, putative, expressed
LOC_Os11g03210	glutathione S-transferase, N-terminal domain containing protein
LOC_Os11g03220	RNA binding protein, putative, expressed
LOC_Os11g03230	nucleoside-triphosphatase, putative, expressed
LOC_Os11g03240	MATE efflux family protein, putative, expressed
LOC_Os11g03260	ligA, putative, expressed
LOC_Os11g03270	nucleoside-triphosphatase, putative, expressed

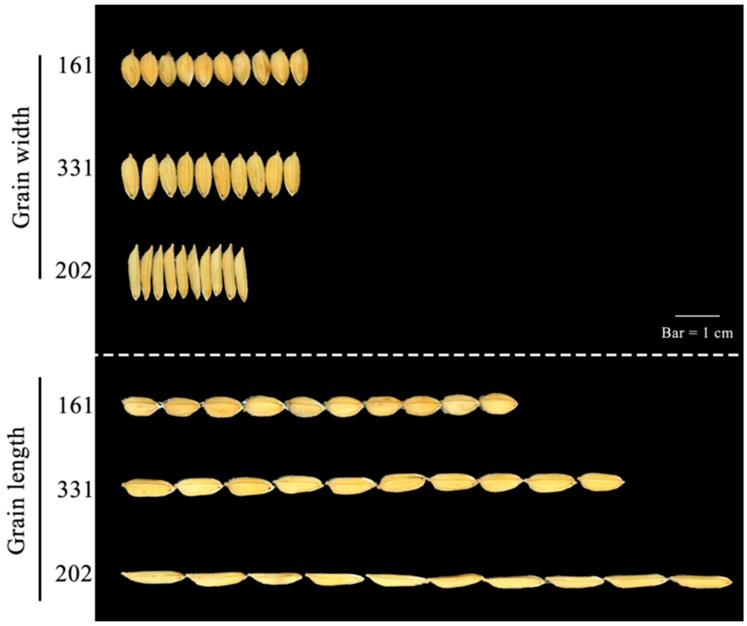

Figure S1. Morphological phenotypic appearance of GL and GW in different plant materials.

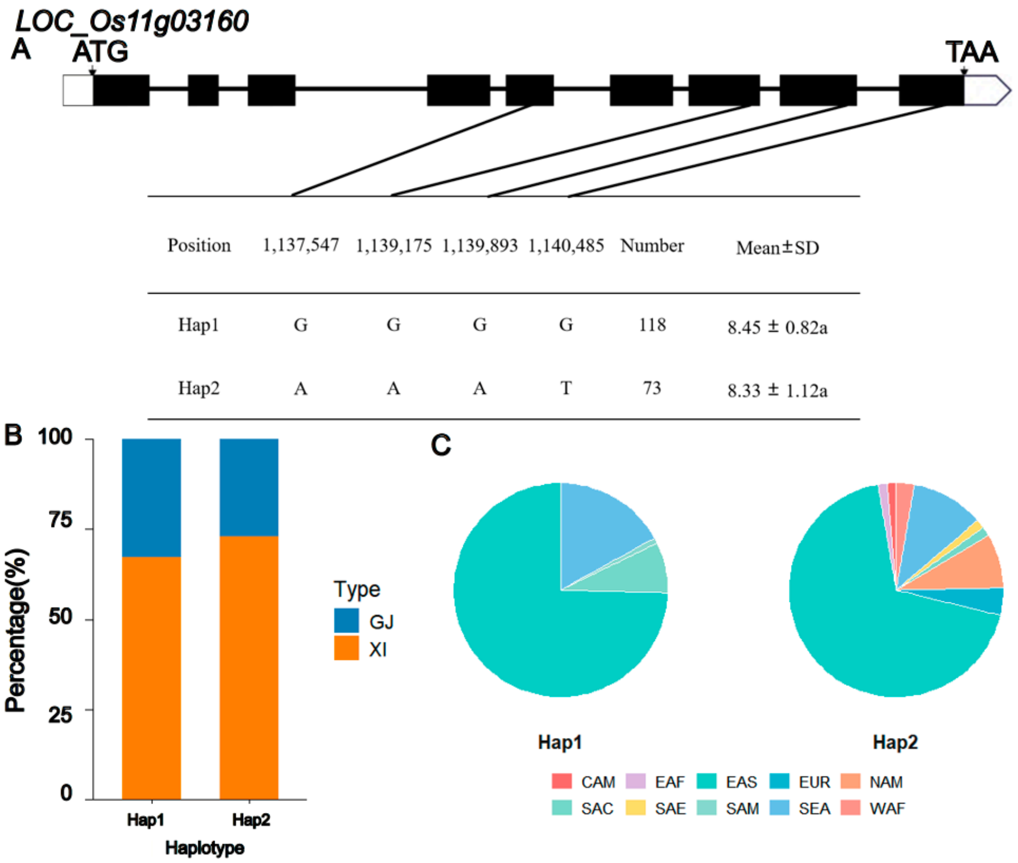

Figure S2. Analysis of the *LOC_Os11g03160* haplotype. (A) Schematic representation of structure and haplotypes. Data are expressed as mean ± standard deviation. Values followed by different lowercase letters (a, b) in the figure indicate significant differences at $P < 0.05$ (tested by Duncan's multiple range test) (B) The subpopulation composition of *LOC_Os11g03160.* (C) Geographical distribution of different haplotypes of *LOC_Os11g03160.*

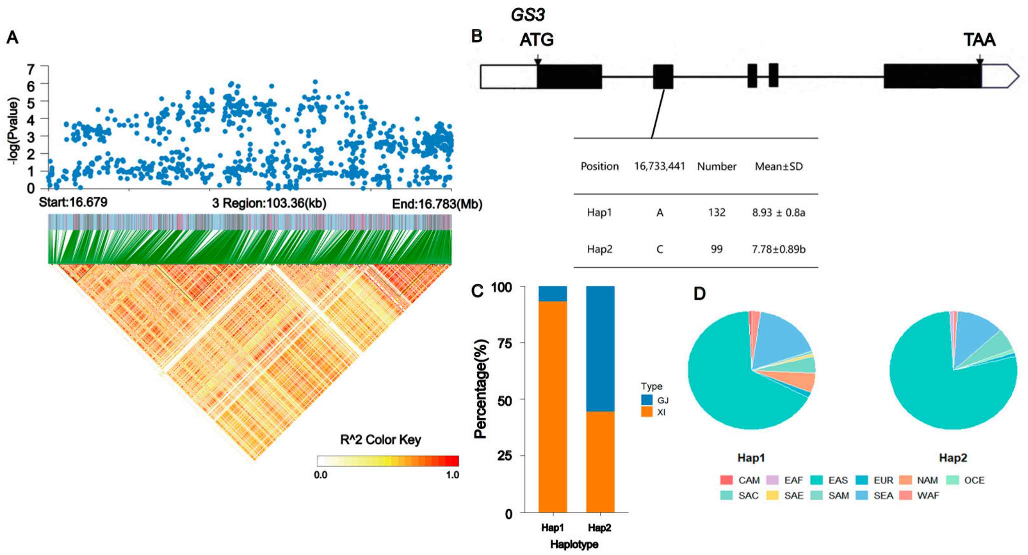

Figure S3. Analysis of the *GS3* haplotype. (A) Linkage disequilibrium plot for SNPs in *GS3*. (B) Schematic representation of *GS3* structure and haplotypes. Different letters indicate significant differences at the 5% level. (This lettering scheme is consistent across subsequent figures.) Data are expressed as mean ± standard deviation. Values followed by different lowercase letters (a, b) in the figure indicate significant differences at $P < 0.05$ (tested by Duncan's multiple range test) (C) The subpopulation composition of *GS3*. (D) Geographical distribution of different haplotypes of *GS3*.

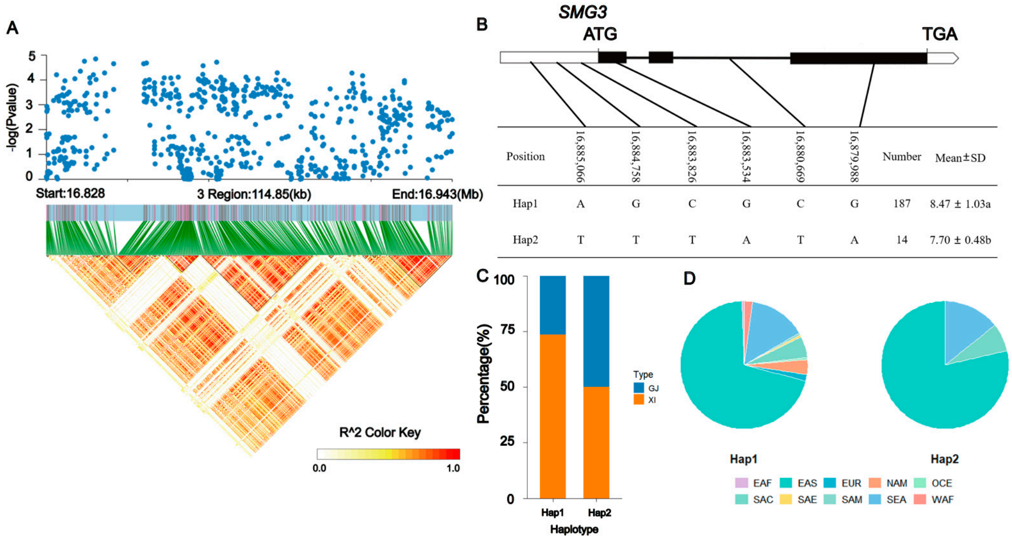

Figure S4. Analysis of the *SMG3* haplotype. (A) Linkage disequilibrium plot for SNPs in *SMG3*. (B) Schematic representation of *SMG3* structure and haplotypes. Data are expressed as mean ± standard deviation. Values followed by different lowercase letters (a, b) in the figure indicate significant differences at $P < 0.05$ (tested by Duncan’s multiple range test) (C) The subpopulation composition of *SMG3*. (D) Geographical distribution of different haplotypes of *SMG3*.

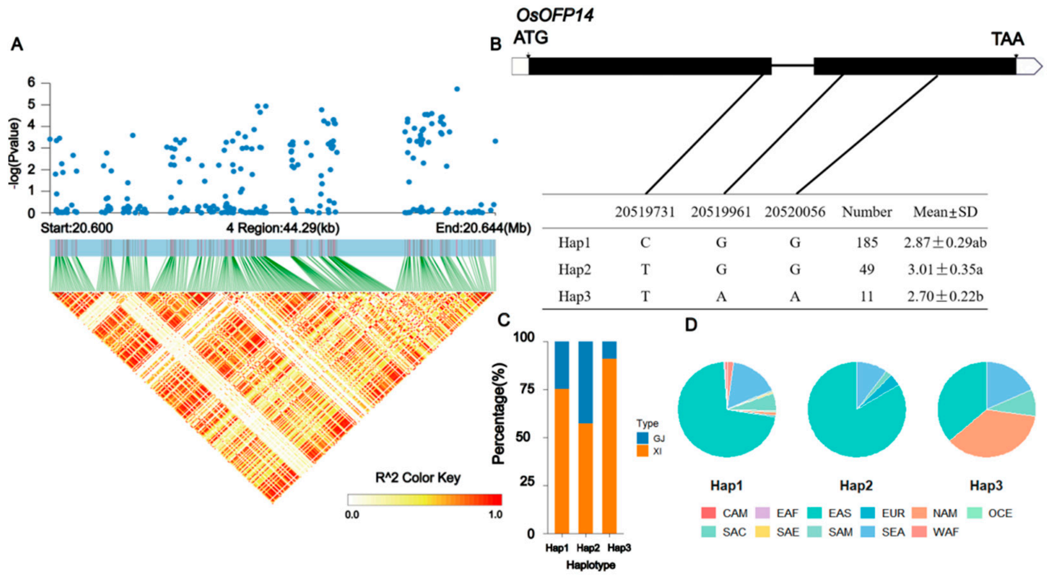

Figure S5. Analysis of the *OsOFP14* haplotype. (A) Linkage disequilibrium plot for SNPs in *OsOFP14*. (B) Schematic representation of *OsOFP14* structure and haplotypes. Data are expressed as mean ± standard deviation. Values followed by different lowercase letters (a, b) in the figure indicate significant differences at $P < 0.05$ (tested by Duncan's multiple range test) (C) The subpopulation composition of *OsOFP14*. (D) Geographical distribution of different haplotypes of *OsOFP14*.
